# Our Bureau of Information

**Published:** 1912-02-17

**Authors:** 


### OUR BUREAU OF INFORMATION.

### THE RESPONSE TO THE CRY OF THE CHILDREN.

Some time ago we published a communication which asked if there was a home which would receive a child whose mother could afford to pay only 3s. 6d. a week. It seemed unlikely that there should be an institution where such a small amount of self-help would be accepted, but in a week or two a reply came. There is such a home. It is in Scarborough. If it were nearer London, one correspondent says, she herself could easily fill it. This means that there are among the working classes many who would gladly give what they can spare from their earnings to obtain for their children the advantages of fresh air, good food, and skilled attendance. We know that a home in a healthy neighbourhood, well managed, and under the care of a trained nurse, cannot be run at the rate of 3s. 6d. a week per inmate; but we offer the example of this home in Scarborough—the "Ida" Home—as a suggestion for the charitable who want to help those who help themselves. Admission to this home is by subscriber's letter, which, if the subscribers are careful to inquire into cases themselves, or to get some responsible person to do so, forms a very reasonable precaution against abuse. And such homes should always be moderate in size, both to ensure personal care of the inmates and to prevent the use of influence which is not based upon personal knowledge of the case. The fact that a parent is willing to pay from 2s. 6d. to 3s. 6d. a week for the care of the child is a guarantee that self-respect and the sense of parental duty are not lacking.

Of course, such a home is directly dependent on charity; it is frankly unremunerative. But we find that it is possible to keep a home—not remunerative, but self-supporting—at from six to eight shillings a week per inmate. Of course, this cannot be done everywhere. It is one of the troubles which meet those who try to help the poor that the poor are very exacting. A distance of a hundred miles seems to them such an enormous way off that they will sooner let a child suffer and die than be parted from it by a two-hours' journey. It is curious to a person of more education or more self-control to hear them reckon, in the case of a person who is convalescent, and only wants a few weeks' fresh air and good treatment to be restored to health, what the cost would be if death ensued at a distance, and what their feelings would be if the (not yet) departed were laid in a grave in alien soil. This sentimentality of the illiterate must always be taken into account. There are places where as good a house can be had for £20 a year as could be rented for £50 in a London suburb, and where, if it is not near a railway station, such boons as good pure milk and fresh eggs, and perhaps even fowls, can be bought for much less than city prices. There are homes which will take the children of the actually poor on these terms.

Suppose a child has recovered from an illness, and ought to go to the country, but family circumstances prevent the mother going with him. In such a case lodgings are out of the question; but a home where the parents could be sure of proper care would be a boon. Such a home can be found at a rate of from 12s. to £1 a week, the price varying according to the locality and to the comforts provided. In either case necessaries would be provided, and in the latter some educational advantages as well. This our correspondence has shown us.

### NEEDS AND HELPERS.

### An Incurables' Home with Industrial Training.

C. L. writes: A Home such as you inquire about in your issue of February 3 is provided in the Home for Cripples in Clapham Park, which will this year be removed to a better situation at Ruislip, near Pinner. The Home was opened in the summer of 1907, and was at first practically a Home for Incurables. An opportunity, however, subsequently presented itself of placing the Home under the direction of a well-known surgeon. This opportunity was not lost, and the Home became a hospital for boys suffering from tuberculosis and other joint diseases, infantile paralysis, etc., provision being made at the same time for the educational and industrial training of the boys during their treatment, which, in many cases, necessarily lasts several years. Workshops are attached, in which the cripples are trained to earn their own living. The success of the Home, as a hospital, has been very marked. Several boys, once seriously deformed, can hardly be recognised as cripples, while others are able to walk who had been bed-ridden for years. The Home is under the care of a Sister of Charity; it is certified by the Local Government Board, and arrangements will soon be made for it to be certified as a special school by the Board of Education. In another Home, St. Vincent's, which is also to be removed to Ruislip this year, the patients are retained till they are twenty years of age.

### Convalescent Home for Young Children.

"Tante" writes: Is there any place where a quite young child can be received without its mother? I mean one too young for the ordinary Children's Convalescent Homes.

(The Invalid Babies' Nursery, Hunstanton, would probably meet "Tante's" requirements. Quite young children are received there, though the sisters who manage the Home prefer them to be over a year old. A small charge is made.)

